# Obsessive-Compulsive Disorder: A Medical School Curriculum and Textbook Review

**DOI:** 10.1177/23821205241242262

**Published:** 2024-03-27

**Authors:** Chelsea A. Lahey, Emily J. Fawcett, Noah Pevie, Rowan B. Seim, Jonathan M. Fawcett

**Affiliations:** Department of Psychology, 7512 Memorial University of Newfoundland, St. John's, NL, Canada

**Keywords:** OCD, medical education, curriculum review

## Abstract

**OBJECTIVES:**

We conducted a curriculum review of Canadian undergraduate medical programs to identify why aggressive obsessions (among those with obsessive-compulsive disorder [OCD]) are so often misidentified by primary care physicians and professional students.

**METHODS:**

This study involved standardized interviews with representatives from Canadian medical schools regarding the content, time, and teaching styles used to deliver curricula related to OCD. Further, we utilized a set of standardized criteria to assess the OCD content of recommended textbooks from these schools.

**RESULTS:**

Canadian medical curricula failed to provide a comprehensive picture of OCD. One-third of medical programs did not provide an example of aggressive obsessions to students, with textbook case examples centered heavily (70%) on contamination or symmetry. Only 25% of programs (and 60% of textbooks) discussed the composition of the Unacceptable Thought Domain to include aggressive, sexual, and religious obsessions. Finally, over half of medical programs failed to indicate that aggressive obsessions are ego-dystonic and do not lead people to harm themselves or others.

**CONCLUSION:**

A series of recommendations are provided for medical schools intended to improve the comprehensiveness of OCD-related training.

## Introduction

Obsessive-compulsive disorder (OCD) has earned its own division in the fifth edition of the *Diagnostic and Statistical Manual of Mental Disorders* (*DSM-5*) where it is characterized by the presence of obsessions (ie, repetitive, unwanted thoughts or impulses causing significant distress), compulsions (ie, mental or behavioral acts that serve to mitigate the distress caused by obsessions), or both.^1^ In those living with OCD, substantial time may be devoted to obsessions or compulsions, causing significant distress or impairment to the individual. Given its time-consuming nature, those with OCD often face significant social and occupational impairment,^2,3^ contributing to a decreased overall quality of life comparable to those living with schizophrenia.^4^ These impairments are further exacerbated by the existing lag between OCD onset and treatment initiation, which has been estimated to take up to 9 years, contributing to the development of lifetime comorbid illness and decreased efficacy of antidepressant medication.^5^

The heterogeneous nature of OCD may contribute to the lag between onset and treatment. For instance, obsessive-compulsive symptoms can be divided into 7 different symptom dimensions: contamination/cleaning, symmetry/ordering/arranging/counting, sexual/religious, aggression, somatic, hoarding/collecting, and miscellaneous obsessions and compulsions.^6^ Contamination and aggressive obsessions are the most common obsessional themes reported in individuals with OCD.^7^ It is critical to note that aggressive obsessions are particularly distressing because they are ego-dystonic, going directly against the individual's true nature, beliefs, and intentions (eg, thoughts of harming one's child as in perinatal OCD).^8,9^ In a recent systematic review, aggressive obsessions were found to be met with significantly more public stigma (eg, perceived dangerousness) than other OCD symptom dimensions,^10^ and desired social distance has been found to be similar for individuals with schizophrenia and aggressive OCD symptoms.^11^ Importantly, individuals with aggressive obsessions are not at heightened risk of harming themselves or others.^12,13^ Therefore, involuntary referral to emergency psychiatric services prompted by a mental health professional with individual and public safety in mind would only serve to falsely confirm that the individual is a true danger. If obsessions are reinforced in this manner, symptoms and outcomes are likely to worsen.^12,14^

Given the almost exclusive focus on contamination and symmetry OCD in popular culture and educational materials,^15-17^ it is perhaps not surprising that health professionals are more likely to misidentify aggressive obsessions in patients than they are contamination and symmetry symptoms. For example, in a series of studies using clinical vignettes, OCD misidentification was found to be highest for aggressive obsessions (over 30%), compared to contamination symptoms (15.8%) among mental health professionals,^18^ with misidentification of aggressive obsessions climbing to as high as 80% for general practitioners.^14^ Similar findings have been observed in students within mental health fields, who were 8 times more likely to misidentify noncontamination OCD than contamination OCD.^19^

General practitioners are often the first point of contact for mental health concerns, including OCD.^20,21^ For instance, in a study of help-seeking among 88 U.K. participants with OCD, the vast majority (73%) went to a GP for help, followed by internet self-help sites (18.2%), and then by seeking out a private therapist or psychiatrist (15.9%).^22^ Underrecognition of OCD in primary care is evidenced by the fact that the incidence and prevalence of OCD in those seeking treatment from general practitioners is 3 times lower than rates obtained through epidemiological studies in the community.^23^ This underrecognition of OCD is likely a result of physician misdiagnosis. Indeed, a recent meta-analysis found that general practitioners struggle to correctly diagnose anxiety disorders when presented with a true case (44.5%), especially without the use of a diagnostic instrument or rating scale.^24^

One explanation for the high rates of OCD misdiagnosis would be that professionals are not trained to identify certain presentations of the disorder. Based on previous work,^19^ it may be the case that medical school curricula focus more heavily on contamination and symmetry presentations. Curriculum reviews represent a method of systematically describing the curriculum of academic programs. Previous curriculum reviews have shown that teaching methods and program content can vary significantly at the local level.^25^ We sought to conduct an OCD-specific curriculum review of medical schools across Canada. We hypothesized that the curriculum would focus disproportionally on contamination and symmetry OCD over other symptom domains, particularly over those featuring aggressive obsessions. We further hypothesized that few medical school programs would explain that individuals with ego-dystonic aggressive obsessions are no more likely than the general population to act upon their intrusive thoughts.

## Methods

### Setting and Sample

An Atlantic Canadian University undertook the evaluation between October of 2020 and February of 2021. A structured questionnaire was created for the purpose of this study, with a representative at each participating medical school completing the questionnaire and providing factual information on the contents of their current medical school curriculum. Nonprobability sampling was utilized, with total population sampling initially employed, as a list of all Canadian University medical schools was created and then a representative from each was contacted. The final sample however was based on availability/volunteer sampling. Under consultation with the local ethics board (ICEHR), because this project was examining program curriculum across Canadian Medical Schools and seeking out factual information about the programs rather than personal opinions, and the individuals contacted were not themselves the focus of the research, the project was exempt from REB review under Article 2.1 of the Tri-Council Policy Statement: Ethical Conduct for Research Involving Humans,^26^ and the requirement of written informed consent was waived.

### Materials and Design

Interview questions are provided in Table 1. Each textbook considered in this study was given a quality rating out of 15 based on standardized questions (see Table 2).

**Table 1.** Standardized interview questions to ask medical program representatives.

1. Where in medical school curriculum is formal teaching related to OCD covered?
2. What year of medical school curriculum is formal teaching related to OCD covered?
3. Roughly how much time (ie, minutes, hours) is devoted to formal teaching about the clinical presentation/differential diagnosis of OCD specifically?
4. What types of educational materials are used to teach about OCD?
5. Would you be willing to share any educational materials you use to teach about OCD?
6. During time in class, are students provided with examples of differing OCD symptom domains or subtypes such as contamination, unacceptable/taboo thoughts, responsibility for harm, and symmetry/need for things to be “just right”?
7. Unacceptable thoughts in OCD include aggressive, sexual, or religious obsessions. Are these 3 touched upon in class?
8. Are medical undergraduates given specific examples of what aggressive obsessions look like (ie, fear of stabbing strangers in public/fearful thoughts of smothering significant other)?
9. Are medical undergraduates taught that aggressive obsessions do not align with the person's beliefs and that there is no increased risk of them following through with their obsessions?

OCD, obsessive-compulsive disorder.

**Table 2.** Questions and corresponding ratings assessing textbook OCD content quality.

Question	Options
1. Overall, how much space covers OCD?	0 = only a paragraph/no stand-alone chapter 1 = sizeable section in another stand-alone chapter 2 = stand-alone chapter
2. Is variety in presenting symptoms/content of obsessions presented?	0 = no description of varying symptom presentations 1 = table or section in text describing varying symptom presentations
3. Does textbook provide information about 4 main symptom domains of OCD? (eg, Abramowitz et al 2010)	0 = 2+ domains missing 1 = missing 1 domain 2 = all domains present 3 = all domains present with examples for each
4. What is the case example/case study focus?	0 = contamination/symmetry only 1 = unacceptable thoughts/responsibility for harm (or covers multiple symptoms including these 2)
5. Does the textbook describe ego-syntonic versus ego-dystonic obsessions?	0 = no 1 = glossary only 2 = yes
6. Is differential diagnosis with other psychiatric conditions reviewed? (eg, OCD vs psychosis)	0 = no 1 = yes
7. How well are aggressive obsessions described?	0 = no description 1 = good description (examples given) 2 = excellent description (gives 3+ examples of the following: aggressive obsessions involving fear of harming oneself, harming others intentionally or accidentally, being responsible for harm, violent images, blurting insults, acting on unwanted impulses, stealing things)
8. Are unacceptable/taboo thoughts thoroughly defined (eg, sexual, aggressive, religious)	0 = no 1 = at least 1 of 3 categories mentioned 2 = all 3 categories mentioned
9. Is risk of violence addressed in the description of aggressive obsessions or OCD generally?	0 = textbook does not make it clear that individuals with aggressive obsessions are unlikely to act on them 1 = textbook makes it clear that individuals with aggressive obsessions are unlikely to act on them

OCD, obsessive-compulsive disorder.

### Procedure

Program representatives involved in providing mental health training to medical students at each of the 17 Canadian medical schools were contacted using a standardized initial recruitment email. No formal inclusion or exclusion criteria were utilized for selecting a program representative, other than familiarity with the OCD training curriculum, thus a number of individuals were contacted ranging from administrators, professors, and psychiatry rotation coordinators. If OCD was covered in both preclerkship and clerkship stages, representatives from both were contacted. No demographic information was collected on program representatives as they were not considered participants as per the nature of the study described above. Interviews were primarily conducted over the phone or via video chat (eg, Zoom). When a representative was unavailable for a live interview, the questions were completed in written format via email. Each interview took 10 to 15 min to conduct. Representatives from the schools were additionally asked to provide any textbook that was either assigned or recommended during a psychiatry rotation.

### Statistical Analysis

Descriptive statistics (eg, frequencies) were used to analyze interview questions and textbook ratings. Results from the interviews and ratings are presented as frequencies (with percentages) for categorical data and as means and standard deviations for continuous data. All statistical analyses were performed using the jamovi Version 2.2.5.^27^

## Results

### Interviews

**Figure 1.**
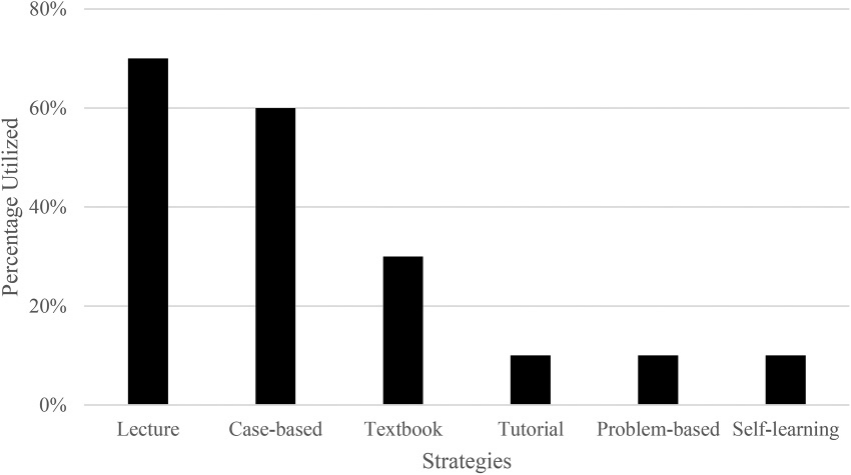
Types of teaching strategies used to disseminate OCD curriculum.

Overall, 9 of 17 (52.94%) Canadian medical schools completed the interview. Over 55% of OCD curricula were distributed across multiple years. OCD-related content was included more often in preclerkship curricula (33.33%) than in clerkship curricula (11.11%). Schools varied in how much time they devoted to teaching about OCD, ranging from 15 min to 2.25 h (*M* = 1.31, *SD* = 0.60).^a^ As depicted in Figure 1, each institution used a different combination of educational approaches, with most using lectures.

OCD, obsessive-compulsive disorder.

Regarding education about the 4 main symptom domains of OCD (ie, Contamination, Symmetry, Unacceptable thoughts, Responsibility for Harm),^28^ all institutions that responded reportedly covered at least 3 of these domains, although this information was unavailable for 2 institutions. There was greater variability across institutions regarding which specific categories of the Unacceptable Thought domain (ie, aggressive, religious, and sexual) were taught, with 33.33% not breaking down this domain by its categories at all, 33.33% making a point to discuss all 3, one program (11.11%) discussing only sexual obsessions, and 22.22% discussing sexual and aggressive obsessions only.

Representatives were asked if they provided a specific example of an aggressive obsession in class. Approximately one-third (33.33%) did not. Critically, over half (55.56%) reported *not* indicating that aggressive obsessions are ego-dystonic, and that those with aggressive obsessions are not at an increased risk of acting on their intrusive thoughts.

#### Textbook Review

Representatives from 14 out of the 17 (82.35%) medical schools responded to our email regarding textbooks. We were provided with 16 textbooks in total. Of these, 4 were excluded for focusing on nonclinical content,^30-33^ and a further 2 were excluded as they were pocketbooks with very limited content.^34,35^ The remaining 10 were separated into 2 categories: comprehensive texts, or those providing a broad overview of relevant knowledge for disorders (*n *= 6), and clinical skill-focused texts, or those covering symptom presentations and clinical conduct (*n *= 4).

The space dedicated to OCD varied across textbooks with 50% featuring a stand-alone chapter. Of those remaining, some texts featured only a brief paragraph within another chapter (20%), while some featured a sizeable section within a chapter not specific to OCD (ie, Anxiety; 30%). Most texts (70%) included a section on differential diagnosis between OCD and other conditions (eg, psychosis).

All textbooks included a table or section recognizing varying OCD symptom presentations. However, many of these texts (40%) did not provide sufficient descriptions of all 4 major symptom domains, with 30% missing at least 2. Of the remaining texts, some included descriptions of all 4 domains (10%) with many instead just including examples of each domain (50%).

Regarding case examples, the majority (70%) of the texts either did not feature a case example at all or featured only case examples for Contamination or Symmetry. The remainder (30%) included case examples of Responsibility for Harm or Unacceptable Thoughts.

As for the categories associated with the Unacceptable Thought domain, the majority (60%) discussed all 3 categories (ie, aggressive, sexual, and religious), while the rest (30%) were missing at least one, with 10% not mentioning any of the categories. The description of aggressive obsessions across texts was moderate. Some did not offer a description at all (10%), others acknowledged their existence briefly (20%), but the majority provided clear examples of aggressive obsessions (70%).

Finally, all but 2 textbooks (80%) failed to include an explicit statement that those who experience aggressive obsessions are at no greater risk to themselves or others. Further, the majority (60%) of textbooks did not describe the difference between ego-syntonic and ego-dystonic thoughts, while 20% described either one or the other but not both, and 20% were sufficient in describing the difference between the 2 within the sections or chapters related to OCD.

The quality ratings across textbooks ranged from 13% to 93% overall. The text that received the highest rating was Lalonde and Pinard.^36^ The highest ratings were given to comprehensive textbooks (*M* = 67.78, *SD* = 22.87), followed by clinical skills texts (*M* = 48.33, *SD* = 23.96).

## Discussion

Our present goal was to assess OCD education in Canadian undergraduate medical programs. It was hypothesized that OCD curricula would focus on Contamination and Symmetry symptomology over other symptom domains (ie, those related to aggressive obsessions). This hypothesis was partially supported. For the interview portion of the study, nearly all representatives reported discussing the 4 major symptom domains in class. However, the depth of these discussions remains in doubt. For instance, while most schools discussed Unacceptable Thought symptoms, only one-third of schools covered all 3 categories of this symptom domain (ie, religious, sexual, and aggressive). A third of the programs did not provide an example of an aggressive obsession. The textbook portion followed a similar pattern of results. All texts mentioned that OCD can vary in presentation; however, Contamination and Symmetry case examples were overrepresented, with only 30% of case examples including Responsibility for Harm or Unacceptable thought symptoms. While the introduction of lesser-known OCD symptoms is positive, without a follow-up description, example, and explanation that thoughts associated pose no risk of action, nor align with the individual's true character and intentions, the danger of misidentifying these symptoms persists.

More importantly, medical school curricula (either course or textual materials) largely failed to differentiate between ego-dystonic and ego-syntonic thinking and critically neglected to mention that those with such ego-dystonic thoughts are at no greater risk of acting out their obsessions than someone without such thoughts: Our interviews found that over half of the schools failed to touch upon these 2 critical elements in their coursework and our textbook review found 2 criteria (ie, ego-syntonic vs ego-dystonic and risk of violence; Table 2) that most texts failed to address (60% and 80% respectively). This lack of coverage is crucial as it is a probable contributor to the misdiagnosis and mishandling by primary healthcare providers of patients with such thoughts. A failure to realize that such patients are unlikely to act out their ego-dystonic thoughts may lead practitioners to take unnecessary action toward those individuals (eg, contacting Child Protective Services regarding a mother experiencing unwanted thoughts of harming her baby), resulting in unnecessary harm to everyone involved, and reinforcing the patient's greatest fear while delaying treatment.^12-14,37^

The lack of exposure to mental health training that medical students receive is not unique to the Canadian landscape but appears to generalize throughout the world. A worldwide survey of medical student psychiatry education found that in 81 out of 83 countries students completed a mandatory psychiatry course. However, great variability existed in the time spent in theory-based and practice-based education (ranging from 1-day to more than 30 days), with more time spent on theory-based teaching than practice-based.^38^ In addition, multiple choice was the most common evaluation method. In terms of medical graduate training, a recent systematic review of medical specialty programs (internal medicine, family medicine, neurology, pediatrics and geriatrics) across 20 countries found that less than 50% of the academic programs included content on mental health.^39^ Further, of the 20 countries, Germany, France, and Croatia excluded context on anxiety disorders in their postgraduate specialty programs. In general, the variability in length of mental health education at the undergraduate level and lack of emphasis on practical teaching combined with a lack of mental health education at the graduate level (and for some countries, direct exclusion of anxiety disorders) sets the stage for the misdiagnosis of OCD.

### What Contributes to the Educational Deficits in OCD?

Divergent ways of classifying obsessive symptoms may contribute to the overemphasis in teaching and learning on more straightforward symptom dimensions such as contamination OCD. Self-report OCD measures vary substantially on the subtypes assessed, from washing, checking, neutralizing, obsessing, ordering, and hoarding on the Obsessive Compulsive Inventory-Revised (OCI-R),^40^ to the Dimensional Obsessive-Compulsive Scale (DOCS),^28^ which maps on to the common symptom dimensions represented in the DSM, including concerns about germs and contamination; concerns about being responsible for harm, injury, or bad luck; unacceptable thoughts; and concerns about symmetry, completeness, and the need for things to be “just right.” Other clinician rated measures such as the gold-standard Yale-Brown Obsessive Compulsive Scale (Y-BOCS)^41^ organizes OCD symptoms into separate categories of obsessions and compulsions, with aggressive, contamination, sexual, religious, symmetry/exactness, somatic, and miscellaneous obsessions placed in distinct categories, and compulsions including cleaning, checking, repeating, counting, and ordering categories. The Y-BOCS also includes hoarding obsessions and compulsions, despite reclassification as a Hoarding Disorder under the DSM-5 OCD and Related Disorders section.^1^

While the existence of several common OCD symptom domains is generally accepted, studies utilizing exploratory factor analysis to distill OCD symptom domains have yielded anywhere from 3 to 6 major symptom domains or factors,^42^ with forbidden or taboo thoughts sometimes further divided into fears of intentional harm (eg, aggressive urges) and pathological doubt (eg, fears of accidental harm).^43,44^ To add further confusion, taboo thoughts were once considered to be “pure obsessions” without associated compulsions, but more recent research suggests that lesser evaluated and more covert (than overt) compulsions such as mental compulsions and reassurance seeking are common in the presence of taboo thoughts of a sexual, aggressive, and/or religious nature.^45,46^ While learners may be unaware of the nuance of conceptualizing primary OCD symptom dimensions, or even the depth of symptoms that can be present within each individual category, these discrepancies may nonetheless contribute to a lack of standardization across curriculum and textbooks in terms of how symptom dimensions are categorized or described.

The current findings argue against there being a scarcity of materials on aggressive symptoms (eg, of the textbooks that described aggressive obsessions, 70% provided clear examples), but that their emphasis varies substantially across institutions and programs (eg, only a third of institutional curricula discussed all 3 specific categories of the Unacceptable Thought domain). This is particularly problematic when textbooks do not offer a description at all of aggressive obsessions, and case examples primarily focus on contamination or symmetry OCD. Given the more intricate nature of unacceptable thoughts compared to the ease of understanding contamination and cleaning obsessions and compulsions due to their representation in popular media^17^ and more everyday transferability (eg, ease of understanding fears of germs post-pandemic), this suggests the further impetus for taking the time to explain (with examples) this particular symptom dimension.

Educational deficits in psychiatry training do not appear to be exclusive to OCD. For instance, a recent meta-analysis assessed the effectiveness of simulation training (eg, role play, simulated patients, etc) for medical students, postgraduate trainees, and medical doctors.^47^ Of the 163 studies identified, the most common clinical topics were alcohol/substance use disorders (80 studies), mood disorders (62 studies), and suicide (36 studies), whereas only 2 studies with medical students included standardized patient interviews portraying OCD. The level of complexity in simulating disorders or areas potentially considered as “subspecialties” appeared to reduce the likelihood of simulation (eg, borderline personality disorder, hallucinations, autism spectrum disorder, dissociative disorders, sexual disorders, etc), suggesting that OCD is not alone in requiring greater emphasis in psychiatry training. Conversely, rather than OCD being considered too complex of a condition to deconstruct, the lack of training may also mirror societal views of OCD that trivialize and depathologize the condition, likening it to a set of personality traits or eccentricities that are positive (“We’re all a little OCD”; “I love having OCD. It makes me really organized”).^48,49^

### Consequences of Misdiagnosis

Unfortunately, misdetection of psychiatric disorders appears to be the norm rather than the exception, with one Canadian study finding misdiagnosis rates as high as 97.8% for social anxiety disorder or 92.7% for bipolar disorder after administering structured diagnostic interviews to over 800 patients across 7 primary care clinic waiting rooms.^50^ The economic burden of medical misdiagnosis is not trivial, with one study estimating conservatively that misdiagnosis of epilepsy for example costs £125 m per year.^51^ Significant long-term consequences can follow misdiagnosis of OCD, such as healthcare professionals contacting child protective services and restricting access to infants in women with perinatal OCD, despite the nearly universal prevalence of harming intrusions in this population and no increased risk of violence.^52,53^ Misdiagnosis can also lead to inappropriate or even potentially harmful medication trials,^51^ such as a case where OCD was misdiagnosed as psychosis and resulted in worsening of symptoms, increased suicide risk, and hospitalization following antipsychotic treatment.^54^

Conversely, underdetection of OCD can result in individuals failing to receive evidence-based treatments, such as antidepressant medication or exposure and response prevention therapy.^55^ On average, it takes nearly 13 years between the onset of OCD symptoms and receiving a diagnosis,^56^ and even then, individuals with OCD rarely receive firstline treatments.^57^ A longer duration of untreated OCD is significantly associated with a higher risk of developing comorbid general medical conditions (eg, metabolic, gastrointestinal, and cardiovascular diseases).^58^ An additional concern with overlooking OCD, is the high prevalence of obsessive-compulsive symptoms, even in cases where full diagnostic criteria are not met. For instance, in a sample of over 2000 patients with anxiety and depressive disorders, nearly a quarter had obsessive-compulsive symptoms, and importantly, the presence of obsessive-compulsive symptoms was related to heightened depression and anxiety symptom severity, greater persistence of symptoms, and greater likelihood of relapse in those with a remitted disorder. These findings suggest viewing comorbid obsessive-compulsive symptoms as a specifier for worsened course and outcomes, requiring greater clinical attention and more intensive treatment.^59^

### What Can Be Done?

Incorporating the socioeconomic, epidemiological, and long-term consequences of misdiagnosis of OCD in primary care into the medical curriculum may help change the landscape of the clinical management of OCD. Education of medical trainees could benefit from the awareness of the main contributing factors to diagnostic errors: inadequate differential diagnosis and overconfidence.^60^ Royce et al^61^ argue for the use of educational interventions such as cognitive bias awareness training (eg, confirmation bias) in medical education to enhance the critical analysis of decision-making and reduce the probability of diagnostic errors. While perhaps more common in residency programs, early introduction of patient safety tools in medical school could help trainees to identify the multitude of factors contributing to diagnostic errors at both the individual and systemic level (eg, modified fishbone diagram).^62^ Further, involving allied health professionals in the diagnostic process is an important part of interprofessional collaboration, which was identified as a key competency for inclusion in health professions education programs.^63^ Future family physicians should also be aware of viable future avenues for rapid psychiatry consultation (eg, rapid eConsult services).^64^

With general practitioners being the greatest prescribers of psychotropic medications (at a greater rate than psychiatrists),^65^ the author of a recent systematic review of misdiagnosis in OCD suggests that more specialized training be provided for those prescribing psychotropic medications.^66^ Any practitioner with diagnosis in their scope of practice may require additional training, including family nurse practitioners who are being viewed as a way to meet the mental health and substance use needs of the population,^67^ but also in fields where enhanced understanding and awareness of OCD could increase referrals to these specialists (eg, certified counselors, sexologists, etc). However, as outlined by Storch,^68^ improving OCD recognition requires curriculum adjustments that extend to other disciplines where the prevalence of OCD exceeds that of the general population (eg, dermatology clinics),^69^ or where there could be a high risk of misdiagnosis or stigmatization (eg, disclosure of harm obsessions in obstetrics).^68^

### Recommendations

Given our results, we believe that greater clarity is needed in how OCD is taught to primary healthcare providers. We hope that bringing such lack of coverage to light, as well as offering recommendations to medical programs may serve to mitigate the issue of OCD misidentification among professionals by targeting their training. To this end, we offer 3 core recommendations:

1. *Separately Describe Each Symptom Domain:* It was found that not all representatives facilitated rigorous discussion (with examples) of the 4 major symptom domains. Therefore, we first recommend lecturers clearly describe and differentiate each symptom domain. Specific mention of the Glazier et al’s^14^ article would bring awareness to the high rate of misidentification that exists for Noncontamination and Nonsymmetry presentations (particularly aggressive obsessions), as well as the risks associated with misidentification. Textbooks should provide examples of symptoms for each major symptom domain, beyond just referencing the heterogeneity of the condition. Case examples presented in class or in textbooks should supply more than one vignette or provide a vignette wherein the character displays symptoms from multiple domains.
  We also recommend exposing students to self-report measures such as the DOCS,^28^ which provides detailed descriptions and examples pertaining to each of the 4 symptom domains and the 3 categories of Unacceptable Thoughts (ie, aggressive, sexual, and religious). Alternatively, it may be helpful to orient trainees to briefer diagnostic screening tools for OCD that could be used efficiently in primary care (such as the 5-item Dimensional Obsessive-Compulsive Scale-Short Form with a suggested cut-off score of 16).^70^
2. *Emphasize that Patients are Unlikely to act on Aggressive Obsessions:* Students must be clearly informed that individuals with aggressive obsessions are unlikely to act on those obsessions, as they are ego-dystonic and highly distressing to those experiencing them.^12^ Due to the overall lack of coverage on risk assessment in those with OCD, we recommend that the article by Veale et al^12^ be discussed. This article outlines how to evaluate whether those with OCD are at risk of harming themselves or others in a variety of scenarios. Discussing this article even briefly could raise awareness regarding the lack of risk in those with aggressive obsessions.
3. *Use Case-based or Problem-based Learning:* The third recommendation is the inclusion of case-based or problem-based learning when teaching about OCD. While not all schools that offered one or the other received perfect scores, the 3 schools that did not utilize either were those that reported not providing specific examples of aggressive obsessions, with 2 failing to describe such thoughts as ego-dystonic as well as the lack of risk associated with them. It has been shown that medical students who apply their skills through case-based or problem-based learning retain significantly more knowledge, take greater responsibility and initiative for their own learning, and demonstrate more engagement in comparison to those who learn through other approaches such as lectures.^71-74^ A recently conducted systematic review and meta-analysis of medical psychology education in China found a large effect size significantly in favor of problem-based learning as opposed to traditional lecture-based teaching in student's final exam scores.^71^ Inclusion of these approaches would also offer the opportunity to learn about lesser-known presentations (eg, aggressive obsessions) in a more practical manner.

Although these recommendations may be interpreted as requiring medical programs to spend a great amount of time on a single subject, our findings suggest that this is not the case. Comprehensiveness did not appear to be dependent on the amount of teaching time. For instance, the program devoting the most time to teaching OCD (ie, 2.25 h) was not the most comprehensive, whereas the program that spent only 15 min on OCD still covered all 4 symptom domains, all 3 of the Unacceptable Thought categories and, while they did not provide an example of aggressive obsessions, they did discuss that the intrusive thoughts associated with OCD are ego-dystonic. Therefore, coverage of important aspects of the clinical presentation and behavioral manifestations of OCD across medical school curricula is not necessarily time-intensive and could result in significant changes in terms of the identification and treatment of individuals with OCD.

### Limitations and Future Directions

There are several limitations of this study. Firstly, while our response rate was fair (52.94% for interviews and 82.35% for textbooks), not all universities responded. Specifically, while we were able to obtain translations of French-only texts, we lacked interview representation from the French-only medical schools that did not take part. This means we do not have an entirely comprehensive picture of OCD curriculum across medical schools in Canada. Additionally, there are schools that teach about OCD at both preclerkship and clerkship levels, but representatives from only one or the other responded in most cases. This is a major limitation as one level may cover OCD more extensively than the other but may not have been represented in this study's results. In addition, the standardized interview questions were created for the purpose of the present study, and therefore were not validated nor pilot-tested prior to the study. Finally, as no inferential statistics were performed, power analyses were not conducted.

Preclerkship and clerkship host different experiences, particularly with the latter involving practical clinical placement wherein students may work with a spectrum of clinical conditions. For instance, while some students may receive detailed instruction on OCD through their placement, others may master a whole other condition. We also only explored OCD education and cannot speak to whether these programs spend a significant amount of time on other disorders. This could be what detracts from OCD coverage; however, as demonstrated by this study, spending more class time on OCD does not necessarily lead to comprehensiveness (ie, discussion around aggressive obsessions and risk). It is also possible that the concept of ego-dystonic versus ego-syntonic thoughts might have been covered outside of the OCD-specific curriculum in a section on general mental health concepts, or in discussion of differential diagnosis for other conditions (eg, psychosis). However, 40% of textbooks described the difference between ego-syntonic and ego-dystonic thoughts in the OCD chapter specifically, which indicates that introducing this concept in the classroom would not be unreasonable. Since over half (55.56%) of institutions reported not indicating that aggressive obsessions are ego-dystonic, and that those with aggressive obsessions are not at an increased risk of acting on their intrusive thoughts, we feel the benefits outweigh the challenges of ensuring the concept is incorporated into psychiatry curriculum, particularly with OCD education. Finally, the current study also did not assess the quality of instruction, which may affect retention and understanding of class material.

Future research could expand this review to medical schools in other countries. Further, it may be interesting to examine what students know about OCD relative to what type of teaching style they have been exposed to (ie, case-based learning, lectures, textbook assignments). Finally, patient engagement in medical education curriculum design is becoming increasingly common, with documented positive outcomes not only for learners (eg, improved communication skills),^75^ but also for the patients themselves (eg, feeling empowered; hoping their contributions lead to better future healthcare experiences).^76^ A recent systematic review of active patient involvement in undergraduate medical education^75^ found that patient participation was linked to numerous learning outcomes such as enhanced patient-centered care, greater understanding of the complexity of the healthcare system, and increases in patient advocacy to name a few. Given that involving patients in healthcare professional training has been associated with improved attitudes toward patients,^77^ including enhanced empathy levels,^78^ this may be particularly salient for psychiatry-related training to help reduce stigmatizing attitudes toward those with mental illness.

## Conclusion

We hope our recommendations inform medical school curricula as it pertains to OCD. With the mindset of quality over quantity, it is not necessary to spend a disproportionate amount of time or text space on the condition. Instead, working to include and connect the key points covered here through citations, discussion, and detailed examples may serve to improve identification rates for the varying presentations of the condition by future medical professionals.

## Notes

a. For 2 programs, we estimated this time as only the time devoted to teaching anxiety more generally was available (eg, 90 min for 6 primary anxiety-related diagnoses, as outlined by Barlow and Ellard,^29^ means 15 min specific to OCD instruction).
